# Two Highly Similar Chitinases from Marine *Vibrio* Species have Different Enzymatic Properties

**DOI:** 10.3390/md18030139

**Published:** 2020-02-27

**Authors:** Xinxin He, Min Yu, Yanhong Wu, Lingman Ran, Weizhi Liu, Xiao-Hua Zhang

**Affiliations:** 1 College of Marine Life Sciences, Ocean University of China, Qingdao 266003, China; hexinxin1995@163.com (X.H.); yumin@ouc.edu.cn (M.Y.); wuyanhong820@163.com (Y.W.); Tendrilia@163.com (L.R.); liuweizhi@ouc.edu.cn (W.L.); 2 Laboratory for Marine Ecology and Environmental Science, Qingdao National Laboratory for Marine Science and Technology, Qingdao 266071, China; 3 Institute of Evolution & Marine Biodiversity, Ocean University of China, Qingdao 266003, China

**Keywords:** chitin, chitinases, *Vibrio*, enzymatic properties, site-directed mutagenesis

## Abstract

Chitinase, as one of the most important extracellular enzymes in the marine environment, has great ecological and applied values. In this study, two chitinases (Chi1557 and Chi4668) with 97.33% amino acid sequences identity were individually found in *Vibrio rotiferianus* and *Vibrio harveyi*. They both were encoding by 561 amino acids, but differed in 15 amino acids and showed different enzymatic properties. The optimal temperature and pH ranges were 45–50 °C and pH 5.0–7.0 for Chi1557, while ~50 °C and pH 3.0–6.0 for Chi4668. K^+^, Mg^2+^, and EDTA increased the enzymatic activity of Chi4668 significantly, yet these factors were inhibitory to Chi1557. Moreover, Chi1557 degraded colloidal chitin to produce (GlcNAc)_2_ and minor GlcNAc, whereas Chi4668 produce (GlcNAc)_2_ with minor (GlcNAc)_3_ and (GlcNAc)_4_. The *K*cat/*K*m of Chi4668 was ~4.7 times higher than that of Chi1557, indicating that Chi4668 had stronger catalytic activity than Chi1557. Furthermore, site-directed mutagenesis was performed on Chi1557 focusing on seven conserved amino acid residues of family GH18 chitinases. Chi1557 was almost completely inactive after Glu154, Gln219, Tyr221, or Trp312 was individually mutated, retained ~50% activity after Tyr37 was mutated, and increased two times activity after Asp152 was mutated, indicating that these six amino acids were key sites for Chi1557.

## 1. Introduction

Chitin, consisting of *β*-(1,4)-linked *N*-acetyl-d-glucosamine (GlcNAc) units, is the most abundant renewable macromolecule organic matter in marine environments [1]. About 10^11^ tons of chitin is produced in the environment each year, and chitin could support nearly 1% of marine bacterial populations [2,3]. Chitin is abundant in the marine seawater, but almost absent in marine sediments, indicating that chitin is rapidly utilized in the upper layers of seawater, where efficient microbial degradation was taking place [4]. Indeed, many marine bacteria could utilize chitin as nutrient source by secreting chitinases, which are the key proteins for chitin degradation [5].

Chitinases (EC 3.2.1.14) degrade chitin by cleaving *β*-(1,4)-glyosidic bonds and releasing mono- and oligomers (i.e., GlcNAc and chitobiose) [6]. According to their hydrolysis properties, chitinases have been classified as exochitinases and endochitinases. Chitin may be cleaved randomly into shorter fragments by endochitinases, whereas exochitinases or chitobiosidase (EC 3.2.1.29) hydrolyze chitin from the terminal end and release GlcNAc or chitobiose [7]. Moreover, chitinases have been categorized into glycoside hydrolase (GH) families 18 and 19, which differ in the amino acid sequences of their catalytic domains and catalytic mechanisms. The chitinases in family GH19 have a high *α*-helical content and hydrolyze chitin using an acid-base mechanism [8]. Family GH19 chitinases are mostly associated with higher plants and some bacterial species including endo-*β*-*N*-acetylglucosaminidase in *Streptomyces griseus* HUT6037, which was the first discovered chitinase in family GH19 [9], and Chi19 from *V. proteolyticus* which was the first characterized GH19 chitinase in *Vibrio* species [10]. The catalytic domains of GH18 chitinases have a (*β*/*α*)_8_ TIM-barrel fold with crucial catalytic residues located on *β*-strand number 4 and contain a diagnostic *DXDXE* motif ending with the catalytic acid [11]. Like ChiA1 from *Bacillus circulans*, its catalytic domain consists of a deep substrate-binding cleft on the top of its (*β*/*α*)_8_-barrel structure [12] and Trp122 and Trp134 on the surface of the catalytic domain proved to be essential for crystalline chitin hydrolysis [13]. In addition, certain amino acid residues affect the catalytic activity of GH18 chitinases by regulating the pka of the catalytic acid (Glu144 and Asp215) and interacting between each other and substrates (Asp142 and Tyr214) [14]. Besides catalytic domains, chitinases often have one or more carbohydrate-binding modules (CBMs), polycystic kidney disease (PKD)-like domain [15], fibronectin III (FnIII)-like domain [16] or other binding modules to improve hydrolysis efficiency. Family GH18 chitinases are widely distributed in bacteria, viruses, plants, fungi, and mammals. Bacteria-derived chitinases belong primarily to the family GH18 [8,17]. To date, many chitinases from various bacteria have been cloned, expressed and characterized, including those from *Vibrio* species [18], *Alcaligenes faecalis* with antioxidant activity [19], *Streptomyces anulatus* showing antifungal and biodegradation properties [20] and *Paenibacillus* with two catalytic domains [21]. 

*Vibrio* species with capability of using chitin as the sole carbon source were important chitin-degrading microorganisms in aquatic environments [22,23]. Till now, many *Vibrio* species were proved able to degrade chitin [22,23]. A previous study found that 37 out of 47 (~80%) of *Vibrio* strains had the ability to degrade chitin [22]. The genome analysis further confirmed the important role of chitin metabolism in Vibrios. In the study of Lin et al. [24], 18 out of 20 *Vibrio* species contain genes chitinase (EC 3.2.1.14) and *β*-*N*-acetylhexosaminidase (EC 3.2.1.52), which could completely hydrolyze chitin to monomer GlcNAc. Like many other chitinolytic bacteria [25,26,27], *Vibrio* species could produce multiple chitinases as a strategy to degrade chitin efficiently. Svitil et al. [28] found 10 chitinases in *V. harveyi*, which produced different enzymes under growth conditions with different chitin substrates. 

In the previous studies, *V. rotiferianus* WXL191 (=*V. rotiferianus* B64D1) was identified as chitin-degrading bacterium based on genomic analysis [24], and *V. harveyi* was concerned as a typical chitin-degrading bacterium [28]. This article described the cloning, expression and characterization of two recombinant chitinases, Chi1557 and Chi4668, individually from *V. rotiferianus* WXL191 and *V. harveyi* WXL538 using *Escherichia coli* (*E. coli*) expression system. Furthermore, the differences in the amino acid composition of the recombinant chitinases and the enzymatic properties of these two chitinases were analyzed and compared. Moreover, to investigate the effect of some key residues in the function of Chi1557, the site-directed mutagenesis was performed.

## 2. Results

### 2.1. Amino Acid Sequences Analysis of Chi1557 and Chi4668

The amino acid sequences identity between Chi1557 (MN555466) from strain *V. rotiferianus* WXL191 and Chi4668 (MN555465) from strain *V. harveyi* WXL538 is 97.33%. These two proteins are both encoding by 561 amino acids with differ in only 15 amino acids (Table S2, Figure S2). Multiple sequence alignment by BLASTP against protein data bank (pdb) database revealed that proteins Chi1557 and Chi4668 shared the highest identities of 60.71%–60.90% and 59.40%–61.01% with the chitinase MmChi60 [29] (PDB id: 4HMC) from *Moritella marina*, respectively. Similar to MmChi60, proteins Chi1557 and Chi4668 are both four-domain structure chitinases annotated by SMART, almost completely overlapping with a TIM *β/α*-barrel without *α+β* insertion at *N*-terminal as catalytic domain, two immunoglobulin-like (Ig-like) domains (pfam DUFs) and a chitin-binding domain (CBM5/12) at the C-terminal (Figure S4). In addition, Chi1557 and Chi4668 were predicted to be extracellular and individually contain an *N*-terminal signal peptide (Table S1). By multiple sequence alignment with CLUSTAL-X, two recombinant chitinases both contain the characteristic motif *DxDxDxE* in their catalytic domain (Figure S5), which is the signature of family GH18 chitinases [11]. The pIs of Chi1557 and Chi4668 are 4.30 and 4.37, and the molecular mass of them are 61.11 kDa and 61.15 kDa predicted with ExPASy database, respectively [30].

Focusing on the different amino acid residues of these two proteins, most of them are hydrophilic in the Chi1557, whereas most of them are hydrophobic in Chi4668. Particularly in the auxiliary domains of these two chitinases, 5 out of 7 different amino acids in the Ig-like domains and the two different amino acids in CBM domain of Chi1557 are both hydrophilic. Whereas these differential amino acid residues of Chi4668 are both hydrophilic except to Arg 470 (Figure S1 and Table S2).

### 2.2. Expression, Purification, and Activity Detection of Recombinant Chitinases

Chitinase-encoding gene *chi*1557 and *chi*4668 were heterologous expressed into *E. coli* BL21(DE3) as an active protein in solute form (the primer pairs were shown in Table 1). 

**Figure 1:**
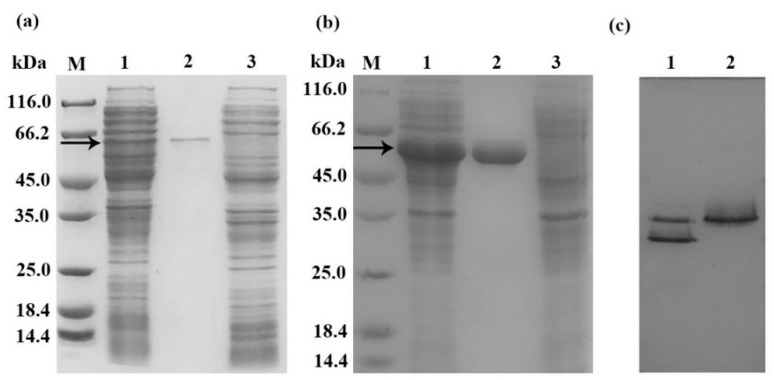
SDS-PAGE and native-PAGE of recombinant chitinases Chi1557 and Chi4668. (**a**) SDS-PAGE of purified Chi1557. M, molecular mass markers; lane 1, cell-free extracts of *Escherichia coli* BL21(DE3)/pET24(+)-*chi*1557; lane 2, purified Chi1557; lane 3, cell-free extracts of *E. coli* BL21 (DE3)/pET24a (+); (**b**) SDS-PAGE of purified Chi4668. M, molecular mass markers; lane 1, cell-free extracts of *E. coli* BL21 (DE3)/pET24 (+)-*chi*4668; lane 2, purified Chi4668; lane 3, cell-free extracts of *E. coli BL21* (DE3)/pET24a (+). (**c**), native-PAGE of Chi1557 and Chi4668. lane 1, purified Chi1557; lane 2, purified Chi4668. Approximately 10 μL of samples were loaded onto each lane and stained by Coomassie brilliant blue. The band indicated by the arrow is the location of the target protein.

The recombinant chitinases were purified by Ni-NTA affinity chromatography with 50–75 mM imidazole. The molecular mass of the purified proteins was estimated as 60–66 kDa by SDS-PAGE (Figure 1), which is consistent with the predicted molecular mass (61 kDa). As the result shown in the native-PAGE (Figure 1), Chi1557 was separated into two distinct bands while Chi4668 was only one band. Here, we speculated that Chi1557 may exist as a dimer protein, while Chi4668 exists as a monomer protein.

The specific activity of recombinant chitinases were observed when using colloidal chitin as substrates at 50 °C. The total enzymatic activity of Chi1557 and Chi4668 were individually 2.05 U and 3.16 U, the total protein content of them were individually 0.13 and 0.18 mg mL**^−^**^1^, and the specific activity of Chi4668 was 41.14 U mg**^−^**^1^ which is higher than that of Chi1557 (23.42 U mg**^−^**^1^) (Table 2). 

### 2.3. The Activity and Stability of Recombinant Chitinases for Temperature and pH 

**Figure 2:**
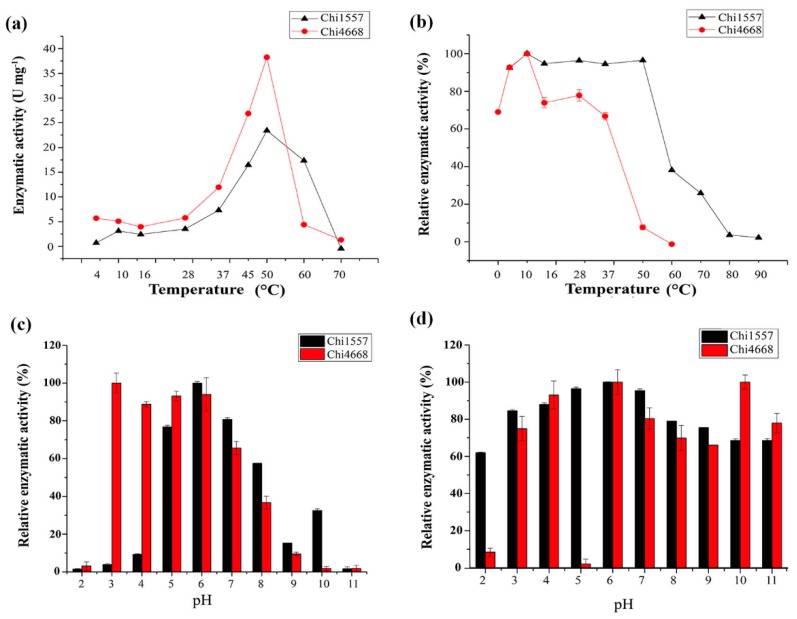
Response of Chi1557 and Chi4668 to temperature and pHs. (**a**) The optimum temperature of Chi1557 and Chi4668. They all show the highest enzyme activity at 50 °C, but the enzyme activity of Chi4668 is about 1.5 times higher than that of Chi1557. (**b**) The temperature stability of Chi1557 and Chi4668. Chi1557 is more stable at temperature between 37 °C to 50 °C. (**c**), The optimum pH of Chi1557 and Chi4668. The optimum pH of Chi1557 is 5.0–7.0, and the optimum pH of Chi4668 is 3.0–6.0. (**d**), the pH stability of Chi1557 and Chi4668.

The optimum temperature of Chi1557 and Chi4668 are both 45–50 °C, but their activities varied greatly at higher temperature (Figure 2). Chi4668 is basically inactivated at 60 °C, while Chi1557 retains about 70% of its activity at 60 °C (Figure 2). For the stability of enzymes, Chi1557 retained more than 90% of its initial activity when incubated at 4–50 °C; the residual activity of the enzyme was reduced by 50% when incubated at 60 °C for one hour. Chi4668 retained only 45% enzymatic activity after incubation at 45 °C, and lost ~90% activity after incubation at 50 °C for an hour. Thus, it was indicated that the temperature stability of Chi4668 is worse than that of Chi1557. Different from Chi4668 and Chi1557, many other chitinases show maximum activities at lower temperatures, including chitinases from the Antarctic psychrotolerant bacterium *Vibrio* sp. Fi:7 (35 °C) [31], *V. furnissii* (35–37 °C) [32], *V. proteolyticus* (40 °C) [10] and *Glaciozyma antarctica* PI12 (15 °C) [33].

Similar with the other chitinases from *Vibrio* species [10,34,35,36,37], Chi1557 showed the highest activity at pH 5.0–7.0 (higher than 70%). It maintained relatively lower activity at pH 8.0 (~60%), pH 9.0 (~20%) and pH 10.0 (~30%), but almost lost its activity at pH 2.0–4.0 (<10%) (Figure 2c). For pH stability, Chi1557 could maintain more than 60% enzymatic activity within a broad range of pH (pH 2.0–11.0) after it was treated in different buffers for 1 h (Figure 2d), indicating that Chi1557 had extremely high pH stability. However, Chi4668 showed the maximum enzymatic activity at pH 3.0–6.0, and lost its activity at pH 9.0–11.0 (<10%) (Figure 2c). For pH stability, Chi4668 have extremely high pH stability and it could maintain more than 70% enzymatic activity within pH 3.0–11.0 except it basically deactivated after treated in 0.1 M citrate buffer at pH 5.0. These observations revealed that Chi1557 prefers alkaline environment whereas Chi4668 prefers acidic environment.

### 2.4. Metal Ions and Reductants on the Activity of Recombinant Chitinases

**Figure 3:**
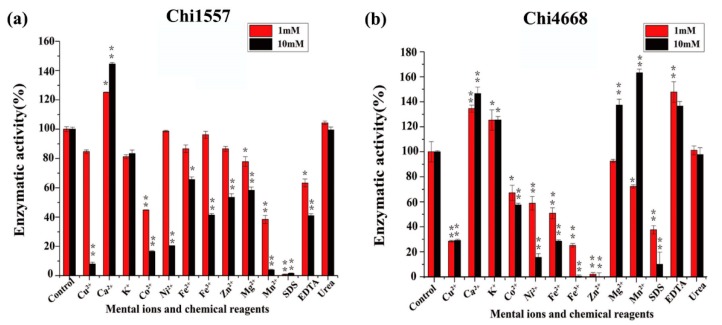
Response of Chi1557 (**a**) and Chi4668 (**b**) to metal ions and reductants. (**a**) Effects of 1 mM and 10 mM metal ions and chemical reagents on the activity of Chi1557; (**b**) Effects of 1 mM and 10 mM metal ions and chemical reagents on the activity of Chi4668. (** *P* < 0.01; * *P* < 0.05).

The effects of metal ions and chemical reagents (EDTA, SDS, and urea) on enzymatic activity were measured (Figure 3). The results show that only Ca^2+^ could increase the activity of Chi1557 up to 125% and 145% of initial activity at concentrations of 1 mM and 10 mM, respectively (Figure 3a). It was in common with chitinases from *Vibrio* sp. Fi:7 [31] and *Sanguibacter antarcticus* [38].

Ca^2+^, K^+^ and EDTA (1 mM and 10 mM) and Mg^2+^ and Mn^2+^ (10 mM) could improve the enzymatic activity of Chi4668 significantly (Figure 3b). 1 mM Cu^2+^, Ni^2+^, Fe^2+^, Fe^3+^, Zn^2+^ and urea had no obvious effect on Chi1557, but significantly inhibited the activity of Chi4668. Similarly, the activities of Chi1557 and Chi4668 were both inhibited with addition of 10 mM Cu^2+^, Co^2+^, Ni^2+^, Mn^2+^, Fe^2+^, Fe^3+^, Zn^2+^, and SDS.

### 2.5. The Kinetic Parameters and Hydrolysis Property of Recombinant Chitinases 

The Michaelis-Menten constant (*K*m) values of Chi1557 and Chi4668 for colloidal chitin are individually 7.94 mg mL**^−^**^1^ and 2.75 mg mL**^−^**^1^. The *K*cat/*K*m values of Chi1557 and Chi4668 for colloidal chitin are 0.40 s**^−^**^1^M**^−^**^1^ and 1.88 s**^−^**^1^M**^−^**^1^, respectively (Table 2).

**Figure 4:**
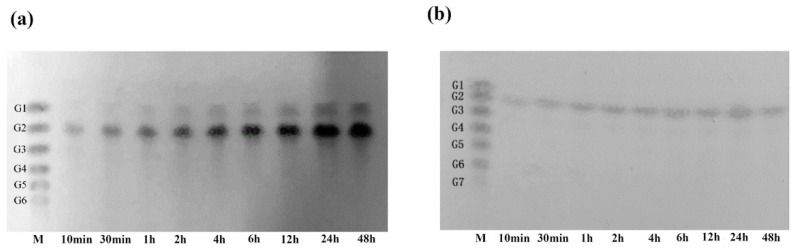
Hydrolysis property for colloidal chitin of Chi1557 and Chi4668. (**a**) The degradation production of Chi1557 for 1% (*w/v*) colloidal chitin; (**b**) The degradation production of Chi4668 for 1% (*w/v*) colloidal chitin. Purified Chi1557 and 1% (*w/v*) colloidal chitin were incubated in 0.1M citrate buffer citrate buffer (pH 5) at 50 °C for different time intervals respectively, Purified Chi4668 and 1% (*w/v*) colloidal chitin were incubated in 0.1 M citrate buffer citrate buffer (pH 6) at 50 °C for different time intervals respectively, and the degradation products were determined by TLC.

The hydrolysis properties of recombinant chitinases on colloidal chitin and *N*-acetyl chitooligosaccharides (COSs) (DP 2-4) were investigated in detail. With the degradation of Chi1557, colloidal chitin was hydrolyzed into (GlcNAc)_2_ with a little GlcNAc (Figure 4a), and (GlcNAc)_3-4_ were hydrolyzed into (GlcNAc)_2_ (Figure S3a), indicating that Chi1557 is an endochitinase. This property was similar to many other chitinases in the family GH18, such as chitinase A from *V. harveyi* as a typical GH18-family chitinase could degrade chitin into (GlcNAc)_2_ [39], chitinases from *V. cholerae* [22], and Pa-Chi from *V. parahaemolyticus* [40]. Differently, the degradation products of Chi4668 were more diverse, it could hydrolyze chitin colloid into (GlcNAc)_2_ with a little (GlcNAc)_3_ and (GlcNAc)_4_ (Figure 4b), hydrolyzed (GlcNAc)_3_ into (GlcNAc)_2_ with GlcNAc and hydrolyzed (GlcNAc)_4_ into (GlcNAc)_2_ with little GlcNAc (Figure S3b). Like Chi4668, the diverse degradation products of Chitinase C1 and Chitinase C3 from strain *V. alginolyticus* H-8 are individually (GlcNAc)_1–3_ and (GlcNAc)_1-6_ [35].

### 2.6. Site-Directed Mutagenesis of Chi1557

**Figure 5:**
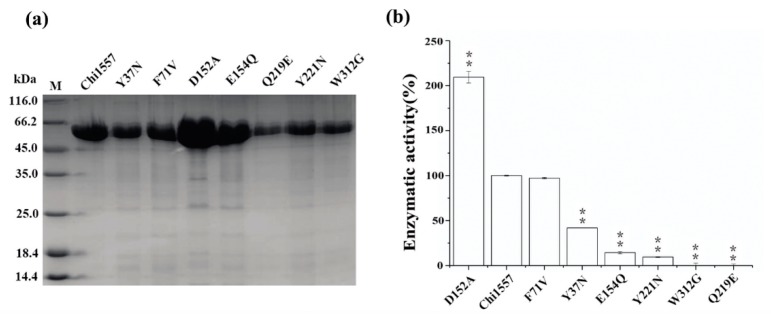
SDS-PAGE and the specific activity of purified chitinases Chi1557 and seven mutant proteins. (**a**), SDS-PAGE of purified Chi1557 and seven mutant proteins. M: marker; 1, purified Chi1557; 2, purified mutant protein Tyr37 (Y37N); 3, purified mutant protein Phe71 (F71V); 4, purified mutant protein Asp152 (D152A); 5, purified mutant protein Glu154 (E154Q); 6, purified mutant protein Gln219 (Q219E); 7, purified mutant protein Tyr221 (Y221N); 8, purified mutant protein Trp312 (W312G). (**b**), The specific activity of Chi1557 and mutant proteins. Chi1557 only retained ~50% of enzymatic activity after mutate Tyr37; its activity was increased twice when mutate Asp152; and Chi1557 could retain original activity after mutate Phe71. (** *P* < 0.01).

Site-directed mutagenesis was performed with Chi1557 to investigate the effect of some key residues in chitinase. Focusing on the conserved sites of chitinases from the family GH18, it has been reported that Asp152 and Glu154 were key sites of the catalytic domain [41]. Trp312 was the key amino acid residue for chitinase binding with chitin [42]. According to the three-dimensional structure of Chi1557 (Figure S4), Gln219 was close to the catalytic center Glu154, and it could maintain appropriately high pKa of catalytic amino acids [14]. Tyr37, Phe71, and Tyr221 were relatively conserved in the other chitinase of GH18 which were also located in the catalytic “pocket” and may keep a different strategy for chitin hydrolysis [42,43,44]. Hence, we selected seven potential key amino residues, Tyr37, Phe71, Asp152, Glu154, Gln219, Tyr221, and Trp312, in Chi1557 for mutation. In this study, we obtain seven purified mutant proteins which show different abilities in chitin degradation. The SDS-PAGE of seven mutant recombinant chitinases and the specific activity of them were tested (Figure 5a). Chitinase Chi1557 was almost completely inactive when Glu154 (mutated to Gln, E154Q), Gln219 (mutated to Glu, Q219E), Tyr221 (mutated to Asn, Y221N) and Trp312 (mutated to Gly, W312G) were mutated; Chi1557 retained ~50% and 100% of enzymatic activity after mutation of Tyr37 (mutated to Asn, Y37N) and Phe71 (mutated to Val, F71V), respectively. Besides, its activity was increased two times when Asp152 was mutated to Ala (D152A) (Figure 5b). It was suggested that Glu154, Gln219, Tyr221, Asp152, Trp312, and Tyr37 are key amino acid residues and absolutely required for Chi1557 activity (Figure 5b).

## 3. Discussion

In this study, two marine *Vibrio* strains were isolated from coastal of China, which could utilize chitin as the sole carbon source for growth and reproduction, indicating that they may play important roles in the marine chitin cycle around its habitat. *V. rotiferianus* WXL191 and *V. harveyi* WXL538 both contained a complete chitin metabolic pathway, including chitin-degrading genes and key transport systems, such as chitinases, glucosamine-1-phosphate *N*-acetyltransferase, *β*-*N*-acetylhexosaminidase and phosphotransferase system (PTS system) (Figure S6). Previous research showed that the genome of *V. rotiferianus* WXL191 (=*V. rotiferianus* B64D1) carried chitinase (EC 3.2.1.14) and *β*-*N*-acetylhexosaminidase (EC 3.2.1.52), which could completely hydrolyze chitin to monomer GlcNAc [24]. Here, two similar chitinases belonged to family GH18, Chi1557 and Chi4668, were obtained from *V. rotiferianus* WXL191 and *V. harveyi* WXL538 and expressed in *E. coli* system. 

Most of *Vibrio*-derived chitinases were classified into family GH18 [24,31,32,37,39,40,45]. In addition to the catalytic domains, these enzymes usually contain one or more chitin-binding domains (ChtBD) [46,47,48] at *N*-terminus and may contain several polycystic kidney disease (PKD)-like domains [32,40,45]. Similarly, chitinases Chi1557 and Chi4668 in this study contained one catalytic domain, one ChtBD and two Ig-like domains, which may help them to improve the chitin degrading efficiency. The molecular mass of *Vibrio*-derived chitinases were between 30–120 kDa, the optimal temperature ranges for most of them were 45–55 °C, and the optimal pH values were between 6.0–8.0 [21,31,32,37,39,40,46,47,48]. However, Chi4668 show maximum enzymatic activity at acidic environment (pH 3.0 and 6.0). The only or main degradation product of these *Vibrio*-derived chitinases in family GH18, including chitinase Pa-Chi from *V. parahaemolyticus* [40] and chitinase A from *V. carchariae* (=*V. harveyi*) [39], was (GlcNAc)_2_. Other chitinases also yielded diverse degradation products, such as the degradation products of chitinase C1 and chitinase C3 from *V. alginolyticus* H-8 were (GlcNAc)_1-3_ and (GlcNAc)_1-6_, respectively [35]. Compared to Chi1557, more diverse degradation products of Chi4668 were identified, including (GlcNAc)_2_ with a little (GlcNAc)_3_ and (GlcNAc)_4_. The enzymatic activity of *Vibrio*-derived chitinases in family GH18 were lower than 10 U mg**^−^**^1^ (ChiA from *Vibrio* sp. Fi: 7, 2–3 U mg**^−^**^1^; chitinase C1 and chitinase C3 from *V. alginolyticus* H-8, 2.8–3.3 U mg**^−^**^1^ and 4.6–5.8 U mg**^−^**^1^), while chitinase from *Vibrio* sp. 11,211 had a higher enzymatic activity (36.5 U mg**^−^**^1^) [31,35,37]. 

In this study, we analyzed and compared the amino acid composition and the enzymatic properties of Chi1557 and Chi4668. Even though the amino acids were much similar (identity is 97.33%), many enzymatic properties of Chi1557 and Chi4668 were different, including the enzymatic activity, the degradation products, and the responses to environmental conditions such as temperature and pH. According to the results of three-dimensional models’ prediction and native-PAGE (Figure 1), Chi1557 may exist as a dimer structure, while Chi4668 exists as a monomer structure. It was reported that the oligomeric structure of enzymes played an important role in biological processes, such as allosteric regulation, conformational stability, and thermal stability [49]. Fraser et al. [50] found that *α*E7 carboxylesterases are more prone to forming dimer or tetramer mutations at high temperatures to improve their stability. Schwab et al. [51] exposited that the monomeric enzyme obtained by polymerization has an enzymatic activity similar to that of the wild-type dimerize. However, its stability was significantly reduced. Hence, we speculated that oligomeric form of Chi1557 may be the reason for its stronger temperature and pH stabilities than Chi4668. Furthermore, a previous study [52] showed that the core domain of the protein containing more hydrophobic amino acids and hydrophobic residues determined the relative positions of secondary structures. Additionally, hydrophobic amino acid residues at key locations were closely associated with the stability of enzymes [53,54]. For *Vibrio*-derived chitinases in GH18 family, the chitin-binding domain is crucial for chitinase-chitin recognition and interactions [48,55]. Compared to Chi557, Chi4668 contains more hydrophobic amino acid residues, especially in the chitin binding domain (Table S1). Previous study [56] has shown that non-conservative substitution of tryptophan residue in chitin-binding domain nearly abolished its chitin-binding affinity. Here, we hypothesized that the hydrophobic tryptophan residues in the chitin-binding domain of Chi4668 may help it maintain the stability of the conformation and facilitate the combination with chitin, and keep high enzymatic activity under extreme conditions (pH = 3.0, 4.0, 6.0).

## 4. Materials and Methods 

### 4.1. Bacterial Strains, Media and Growth Conditions 

*V. rotiferianus* WXL191 (=*V. rotiferianus* B64D1) was isolated from the bottom water (17.5 m water depth) of Bohai Sea at 119.04°E, 38.23°N during the expedition on the R/V *Dong Fang Hong* 2 in August 2015 [57]. *V. harveyi* WXL538 was obtained from the East China Sea (at water depth of 25 m) at 122.56°E, 31.35°N during the expedition on the R/V *Dong Fang Hong* 2 in October 2015 [24]. Both strains were isolated using thiosulfate citrate bile salts sucrose (TCBS) agar (Hopebio, Qingdao, China) and demonstrated strong capacity to degrade chitin when growing on chitin agar plates. Then, these two purified strains were cultured on marine agar 2216E (Hopebio, Qingdao, China) plates at 28 °C for further research. The complete genome sequences of *Vibrio rotiferianus* WXL191 (=*V. rotiferianus* B64D1) and *V. harveyi* WXL538 have been deposited in NCBI GenBank server under the accession number CP018311 to CP018312 and CP045070 to CP045071, respectively. And *E. coli* BL21(DE3) was cultured on Luria-Bertani (LB) agar at 37 °C, and used as a host for expressing proteins whose encoding genes were cloned into pET24a (+) (Novagen, Beijing, China).

### 4.2. Sequence Analysis of Chitinase Genes

The extraction of total genomic DNA, prediction and annotation of chitinase genes were based on the methods of Lin et al. [24]. The bioinformatic analyses of chitinase sequences as follows: the amino acid sequences of chitinases were analyzed by BLASTP against protein data bank (pdb) database (https://blast.ncbi.nlm.nih.gov/) [58]; the closely related chitinases of Chi1557 and Chi4668 were obtained from NCBI, phylogenetic relationships between Chi1557, Chi4668, and the closely related chitinases were constructed using MEGA version 7.0 [59]; the three-dimensional models were predicted using SwissModel (https://www.swissmodel.expasy.org/interactive) and analyzed by PyMOL [60]; the Molecular mass and pI of chitinases were predicted by ExPASy database (https://web.expasy.org/compute_pi/) [30] and the signal peptide was predicted by the SignalP 4.1 server (http://www.cbs.dtu.dk/services/SignalP/) [61]. The GenBank accession number for the chitinase gene sequences of *chi*1557 and *chi*4668 are MN555466 and MN555465, respectively.

### 4.3. Preparation of Chitin Colloids

Colloidal chitin was prepared from commercial chitin by the method of Roberts and Selitrennikoff [62] with some modifications. Briefly, the chitin powder (Sigma-Aldrich, C7170, Munich, Germany) was rinsed in 1 mol L**^−^**^1^ HCL and 1 mol L**^−^**^1^ sodium hydroxide solutions five times in turn (about 2 h each time). And then the deposit was washed 4 times immersing in 95% ethanol, and then dry naturally. Sixty milliliters of concentrated HCl was added slowly into 5 g of processed chitin powder and left at 4 °C with vigorous stirring (about 4–5 h). The mixture was adjusted to pH 7.0 with 10 mol L**^−^**^1^ sodium hydroxide and washed with 5 liters of ice-cold deionized water. The precipitant was collected by centrifugation at 5000 g for 10 min at 4 °C, and the colloidal chitin solution (5%) was prepared and stored at 4 °C until further applications.

### 4.4. Expression and Purification of Recombinant Chitinases

To obtain recombinant proteins without signal peptide, the putative gene was amplified with the primer pairs Chi1557F-Chi1557R, Chi4668F-Chi4668R (Table 1). The genomic DNA of strains WXL191 and WXL531 were used as templates for polymerase chain reaction (PCR) amplification of chi1557 and chi4668, respectively. The expression and purification of chitinases in *E. coli* BL21(DE3) were performed according to Tang et al. [63]. The purified recombinant chitinases were assessed by 12% sulfate-polyacrylamide gel electrophoresis (SDS-PAGE) according to the method of Laemmli et al. [64] and nondenaturing conditions PAGE (native PAGE) according to the method of Davis et al. [65].

### 4.5. Chitinase Activity Assay and Protein Quantification

Chitinase activity was detected by the methods of Lee et al. [66] with modification. Briefly, 190 μL of 1% (*w/v*) colloidal chitin was incubated at 50 °C for 10 min, then 10 μL purified enzyme was added with continued incubation at 50 °C for 30 min. The reducing sugars released were determined by the modified dinitrosalicylic acid (DNS) method [67]. One unit (U) of chitinase activity was defined as the amount of enzyme that released 1 μmol of reducing sugars per minute in 1 mL reaction mixture under the assay conditions by using GlcNAc as the standard. Protein concentration was measured by the Bradford method [68] using bovine serum albumin (BSA) as the standard.

### 4.6. Characterization of Purified Chitinases

The optimal pH of Chi1557 and Chi4668 were measured between pH 2.0–11.0 (at intervals of 1.0) using four kinds of buffer systems: 0.05 M Glycine-HCl (pH 2.0–4.0), 0.1 M citrate (pH 5.0–7.0), 0.05 M Tris-HCl (pH 8.0 and 9.0), and 0.05 M Glycine-NaOH (pH 10.0 and 11.0). To determine the pH stability of the chitinases, the purified enzyme samples were incubated in the above-mentioned buffers at 4 °C for 1 h. And the residual activities were tested at 50 °C. The optimal temperature of chitinases was studied by incubating the enzyme samples with substrate in optimal pH at 4, 10, 16, 28, 37, 45, 50, 60, and 70 °C. For detecting the thermostability, the enzyme samples were incubated in optimal pH at 4, 10, 16, 28, 37, and 50–90 °C (at interval of 10 °C) for 1 h, and then the residual activities were tested. To study the effects of metal ions and chemical reagents (EDTA, SDS and urea), the enzymatic activity was measured by standard methods in the presence of Na^+^, K^+^, Ca^2+^, Fe^3+^, Mn^2+^, Al^3+^, Co^2+^, Ni^2+^, Fe^2+^, Cu^2+^, Mg^2+^, Zn^2+^, and chemical reagents (EDTA, SDS, and urea) at final concentration of 1 mM and 10 mM. Also, the residual activities were tested.

### 4.7. Kinetic Parameters and Hydrolytic Properties of Chitinases

The kinetic parameters of chitinases in colloidal chitin was determined by measuring enzymatic activity at 50 °C in optimum pH for 30 min with substrate concentrations of 0.05%, 0.1%, 0.2%–0.8% (at interval of 0.2%), 1.0%, and 2.0%. The *K*m and *V*max values were calculated from kinetic data fitting of the Michaelis–Menten equation [69]. 

The hydrolysis products of chitinases were determined by thin layer chromatographic (TLC) method using colloidal chitin and (GlcNAc)_2-4_ as substrates [70]. Briefly, purified chitinase and 1% (*w/v*) colloidal chitin or (GlcNAc)_2-4_ were mixed in optimum pH, and then the mixtures were incubated at optimum temperature (50 °C) for 10 min, 30 min, 1 h, 2 h, or 4 h. The reaction products were spotted onto a Silica gel plate (Merck, Damsladt, Germany), and spread using butanol: water: ammonia water (6:3:1, *v/v/v*) as spreading agent. The plate was sprinkled with chromogenic agent (containing 4 g diphenylamine, 4 mL aniline, 2 mL concentrated hydrochloric acid, 20 mL phosphate, and 200 mL acetone). 

### 4.8. Site-Directed Mutagenesis of Chi1557

Site-directed mutagenesis was used to determine the effects of several amino acid residues on enzyme activity of Chi1557. Here, based on the previous studies on key amino acid residues [13,42,71,72,73] and protein structure of family GH18 chitinases [59], as well as the predicted structure of protein Chi1557 in this study, we intended to alter the key amino acid residues Tyr37 by Asn, replace Phe71 by Val, replace Asp152 by Ala, replace Glu154 by Gln, replace Gln219 by Glu, replace Tyr221 by Asn, and replace Trp312 by Gly, respectively. The plasmid pET24a (+)-*chi*1557 was used as PCR template, and primers used to obtain each mutant are listed in Table 1. Positive mutations selected by cloning were confirmed by DNA sequencing. Each mutated protein was expressed and purified to measure its chitin-degrading activities, as described above. 

## Figures and Tables

**Table 1:** Primers used in cloning Chi1557, Chi4668, and the mutagenesis of Chi1557. Primers were designed using the Primer-primer 5 design tool. The cleavage sites are underlined, and the mutagenic nucleotides are represented in lowercase.

Primer	Sequence (5’-3’) b	Restriction Site and Mutant Amino Acid
Chi1557-F	5-CGCGGATCCATGAATGAGATGGTGA-3	BamH I
Chi1557-R	5-TAGCTCGAGCAACTTATCCCACGCG-3	Xho I
Chi4668-F	5’-CGCGGATCCATGAATGAAATGGTGA-3’	BamH I
Chi4668-R	5’-CCCAAGCTTCAACTTATCCCATGCG-3’	Hind III
Y37N-F	5’-GTAGTCGGT***a***ATTGGCATAACTGGT-3’	Tyr37
Y37N-R	5’-GACACCACTATCTGGATTCACCATC-3’	
F71V-F	5’-AACGTCTCC***g***TTATGAAGGTGT-3’	Phe71
F71V-R	5’-AACCACATTGTACATAGGATCAACT-3’	
D152A-F	5’-GGTCTGGACATCG***c***CTTAGAGCA-3’	Asp152
D152A-R	5’-ATCAAAGCCGAACTTGTCAGTCAGG-3’	
E154Q-F	5’-GACATCGACTTA***c***AGCAATCTGCAG-3’	Glu154
E154Q-R	5’-CAGACCATCAAAGCCGAACTTGT-3’	
Q219E-F	5’-ATCAACCCT***g***AATTTTACAACCAAG-3’	Gln219
Q219E-R	5’-CCAATCGTAGTACCCTTCTAATCCA-3’	
Y221N-F	5’-CCTCAATTT***a***ACAACCAAGGTGG-3’	Tyr221
Y221N-R	5’-GTTGATCCAATCGTAGTACCCTTCT-3’	
W312G-F	5’-GTAATGACA***g***GGTCGGTGAACTGGG-3’	Trp312
W312G-R	5’-GCCACGAAGTGCCTGCCCTTG-3’	

**Table 2:** The enzymology properties of Chi1557 and Chi4668.

Enzymology Properties	Chi1557-Ni	Chi4668-Ni
The optimal temperature (°C)	45–50	~50
The optimal pH	5.0–7.0	3.0–6.0
Total enzymatic activity (U)	2.05	3.16
Total protein content (mg mL^−1^)	0.13	0.18
Specific activity (U mg^−1^)	23.42	41.14
The kinetic parameters:		
*V*max (mg U^−1^)	2.94	6.21
*K*m (mg mL^−1^)	7.94	2.75
*K*cat (s^−1^)	3.00	5.18
*K*cat/*K*m (s^−1^M^−1^)	0.40	1.88

## Supplementary Materials

The following are available online at https://www.mdpi.com/1660-3397/18/3/139/s1. Supplementary data of this work can be found in online version of the paper, Figure S1: Neighbor-joining phylogenic tree based on amino acid sequences of Chi1557, Chi4668 and other chitinases from bacteria was built with CLUSTAL-X in the MEGA version 7.0, Figure S2: Multiple sequence alignment of the amino acid sequences of Chi1557 and Chi4668 by CLUSTAL-X in the MEGA version 7.0, Figure S3: The degradation productions of Chi1557 and Chi4668 for N-acetyl COSs (DP 2-4), Figure S4: Three-dimensional structure of Chi1557, Figure S5: Multiple sequence alignment of the amino acid sequences of Chi1557, Chi4668 and other family GH18 chitinases from different bacterial by MUSCLE program in the MEGA version 7.0 and enhanced by ESPript v3.0, Figure S6: The chitin metabolic pathway in *Vibrio* rotiferianus WXL191 and *V. harveyi* WXL538 annotated by KAAS (KEGG Automatic Annotation Server). Table S1: The confidently predicted domains, repeats, motifs and features of Chi1557 and Chi4668, Table S2: The different amino acids of chitinases Chi1557 and Chi4668.

## Abbreviations

aa, amino acid; CBMs, carbohydrate-binding modules; ChtBD, chitin-binding domains; COSs, chitooligosaccharides; FnIII, fibronectin III; GH, glycoside hydrolase; Ig-like, immunoglobulin-like; PKD, polycystic kidney disease; SDS-PAGE, sulfate-polyacrylamide gel electrophoresis; TLC, thin layer chromatography.
