# Supplementary material for: Two Highly Similar Chitinases from Marine Vibrio Species have Different Enzymatic Properties
Source: Mar Drugs. 2020 Feb 27;18(3):139. doi: 10.3390/md18030139 (PMC7143101; doi:10.3390/md18030139)
Supplement: Supplementary file 1 [file marinedrugs-18-00139-s001.pdf]

Supplementary material for

Two highly similar chitinases from marine *Vibrio* species have different enzymatic properties

Xinxin He[1†], Min Yu[1†], Yanhong Wu[1], Lingman Ran[1], Weizhi Liu[1] and Xiao-Hua Zhang [1,2,3,*]

[1] College of Marine Life Sciences, Ocean University of China, Qingdao 266003, PR China; hexinxin1995@163.com(X.H.); yumin@ouc.edu.cn(M.Y.); wuyanhong820@163.com(Y.W.); Tendrilia@163.com(L.R.); liuweizhi@ouc.edu.cn(W.L.)

[2] Laboratory for Marine Ecology and Environmental Science, Qingdao National Laboratory for Marine Science and Technology, Qingdao 266071, China;

[3] Institute of Evolution & Marine Biodiversity, Ocean University of China, Qingdao 266003, China

* Correspondence: xhzhang@ouc.edu.cn(X-H.Z.); Tel.: +86-532-82032721(X-H.Z.)

[†] These authors contributed equally to this work.

Received: date; Accepted: date; Published: date

Abbreviations:

GH, glycoside hydrolase; Ig-like, Immunoglobulin-like; aa, amino acid; COSs, chitooligosaccharides; ChtBD, chitin-binding domain; Glyco_18, hydrolytic domain of the enzymes in family GH18

Six figures and two tables were shown in the supplemental materials.

Table S1. The confidently predicted domains, repeats, motifs and features of Chi1557 and Chi4668.

predicted domains, repeats, motifs and features:	Chi1557	Chi4668
Signal peptide	1-23aa	1-23aa
Glyco_18	33-317aa	32-317aa
Ig-like domin	352-424aa	353-424aa
Ig-like domin	429-501aa	429-501aa
Pfam: Big_3	433-496aa	433-496aa
ChtBD3(CBM 5/12)	507-557aa	507-557aa

Table S2. The different amino acids of chitinases Chi1557 and Chi4668.

hydrophilic amino acid (*), hydrophobic amino acid (-), acid amino acid (a), basic amino acid (b), neutral amino acids (n), unknown (/).

No.	site	Chi1557	Chi4668
1	31	S (* n)	G (/ n)
2	60	V (- n)	I (- n)
3	103	G (/ n)	S (* n)
4	281	A (* n)	V (- n)
5	321	D (* a)	N (/ n)
6	326	E (- a)	A (- n)
7	354	L (* n)	F (- n)
8	358	S (* n)	N (/ n)
9	367	S (* n)	A (- n)
10	431	F (- n)	L (- n)
11	447	P (- n)	L (- n)
12	470	S (* n)	R (* b)
13	486	D (* a)	N (/ n)
14	515	T (* n)	A (- n)
15	552	T (* n)	V (- n)

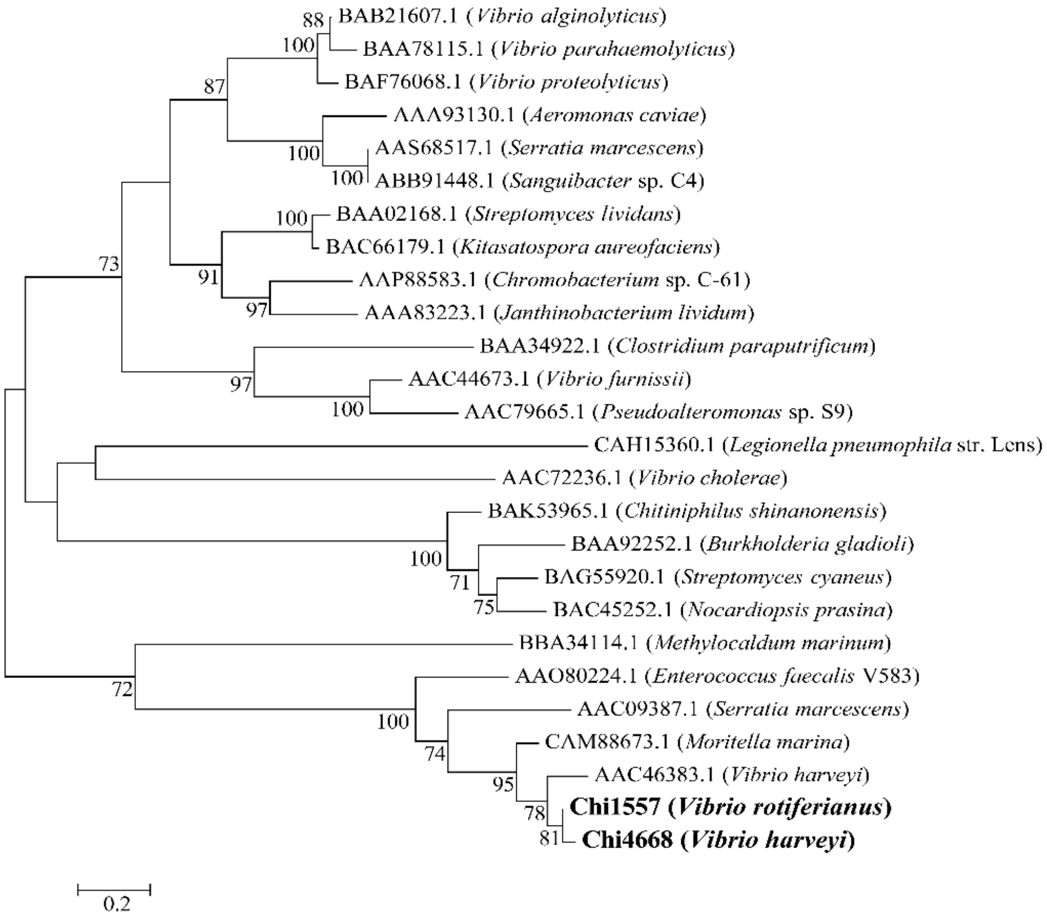

Figure S1. Neighbor-joining phylogenic tree based on amino acid sequences of Chi1557, Chi4668 and other chitinases from bacteria was built with CLUSTAL-X in the MEGA version 7.0. Bootstrap values were calculated based on 1,000 replicates. Bar, 0.2 substitutions per nucleotide position. All amino acid sequences, which belonged to family GH18 and family GH19 were from CAZy database.

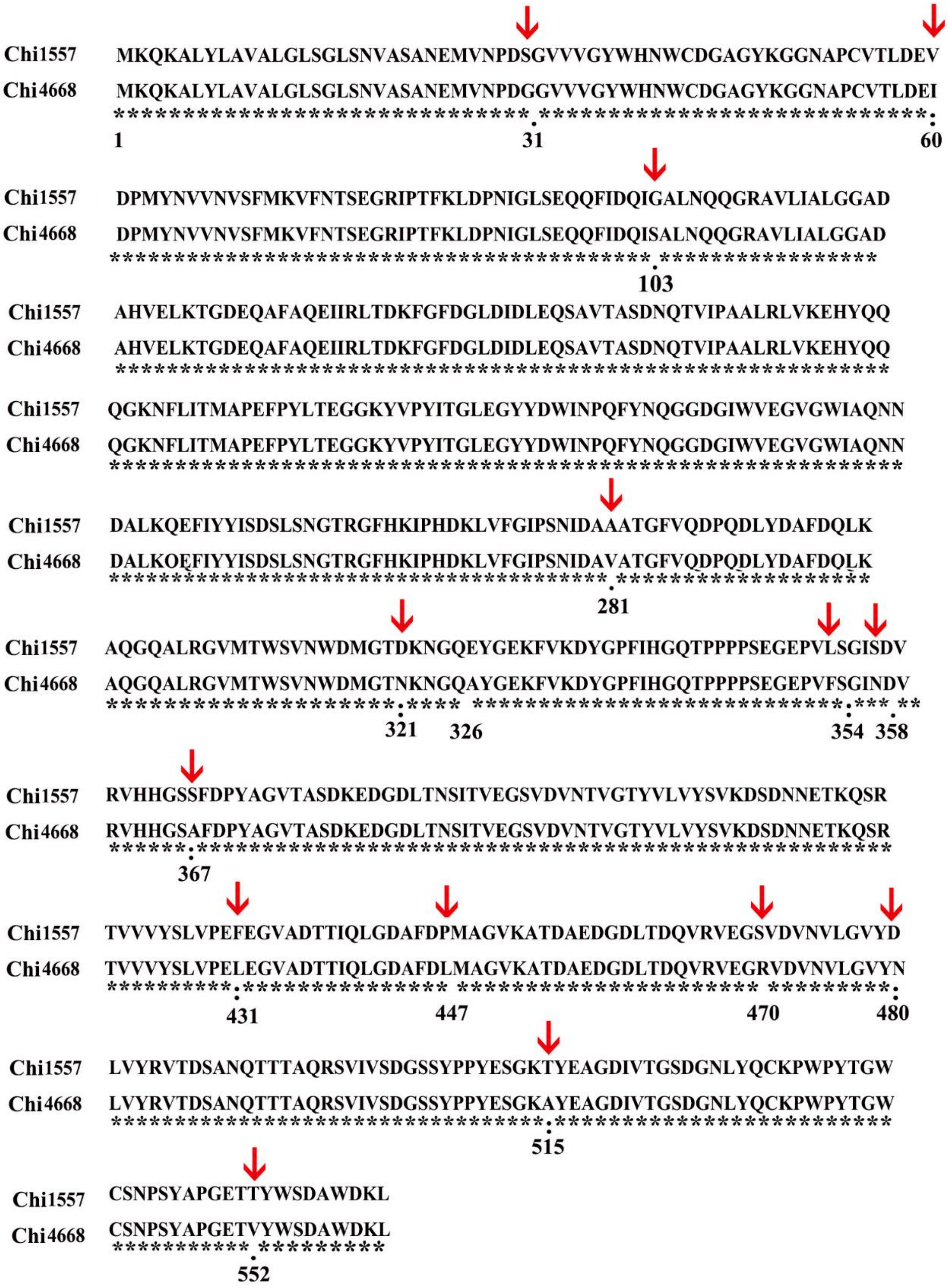

Figure S2. Multiple sequence alignment of the amino acid sequences of Chi1557 and Chi4668 by CLUSTAL 2.1 multiple sequence alignment (https://www.genome.jp/tools-bin/clustalw). The arrows at positions corresponding to the exchanged amino acids.

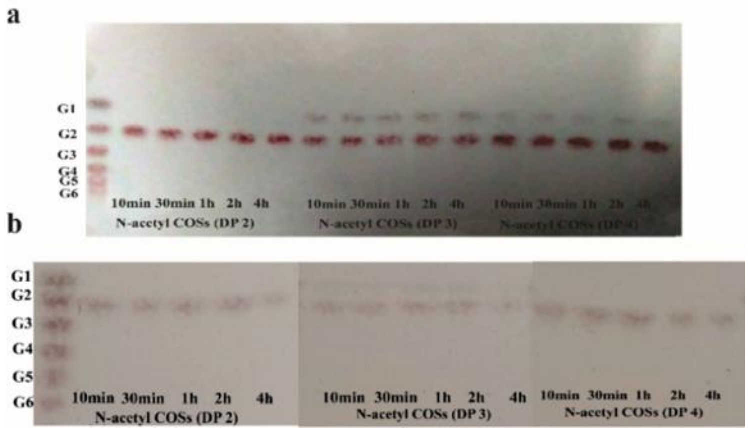

Figure S3. The degradation productions of Chi1557 and Chi4668 for *N*-acetyl COSs (DP 2-4). a, the degradation productions of Chi1557 for *N*-acetyl COSs (DP 2-4); b, the degradation productions of Chi4668 for *N*-acetyl COSs (DP 2-4). Purified Chi1557 and 1% (w/v) of colloidal chitin were incubated in 0.1M citrate buffer citrate buffer (pH 5) at 50 °C for different time intervals respectively. Purified Chi4668 and 1% (w/v) of colloidal chitin were incubated in 0.1M citrate buffer (pH 6) at 50 °C for different time intervals respectively, and the degradation productions were determined by TLC.

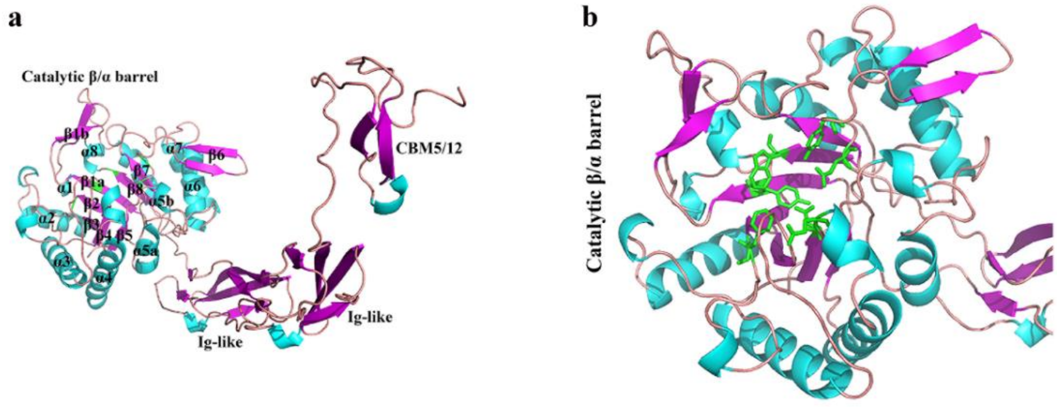

Figure S4. Three-dimensional structure of Chi1557 predicted with SwissModel. a, Three-dimensional structure of Chi1557; b, Three-dimensional structure of catalytic domain. α-helix, β-sheet and amino acids which will be mutated point were marked with blue, purple and green, respectively.

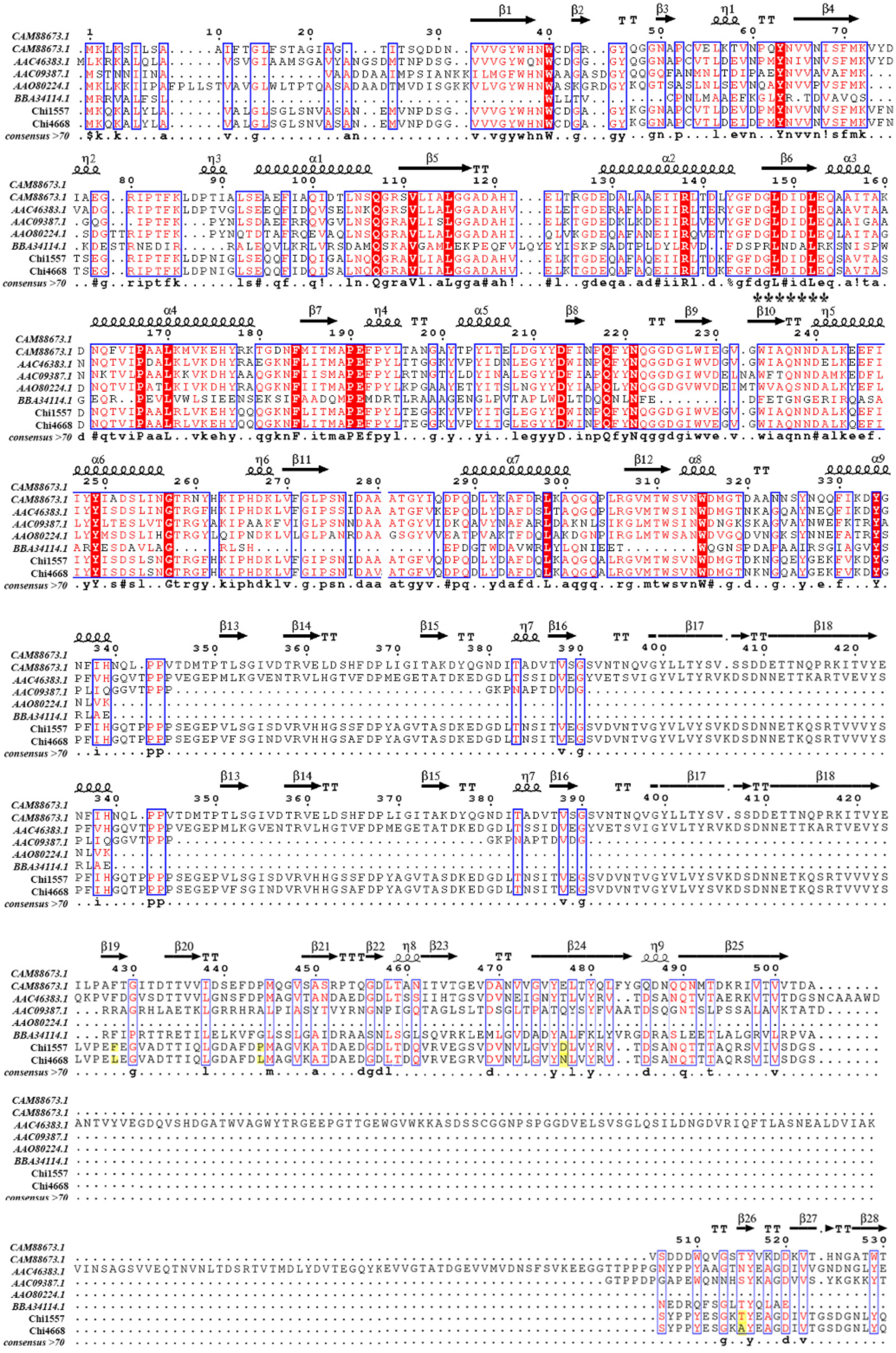
β1 β2 TT β3 η1 TT β4
1 10 20 30 40 50 60 70
CAM88673.1 .MKLKSILSA.....AIFTGLFSTAGIAG...TITSQDDN..VVVGYWHNWCDGR..GYQGGNAPCVELKTVNPQYNVVNISFMKVYD
AAC46383.1 MLKRKALQLA.....VSVGIAAMSGAVYANGSDMTNPDSG..VVVGYWQNWCDGG..GYQGGNAPCVTLDEVNPMYNIVNVSFMKVYD
AAC09387.1 .MSTNNIINA..............VAADDAAIMPSIANKKILMGFWHNWAAGASDGYQQGQFANMNLTDIPAEYNVVAVAFMK...
AAO80224.1 .MKLKKIIPAFPLLSTVAVGLWLTPTQASADAADTMVDISGKKVLVGYWHNWASKGRDGYKQGTSASLNLSEVNQAYNVVPVSFMK...
BBA34114.1 .MRRVALFSL.....WLLTV.......CPNLMAAEFKGLYR.TDVAVQS...
Chi1557 .MKQKALYLA.....VALGLSGLSNVASAN..EMVNPDSG..VVVGYWHNWCDGA..GYKGGNAPCVTLDEVDPMYNVVNVSFMKVFN
Chi4668 .MKQKALYLA.....VALGLSGLSNVASAN..EMVNPDGG..VVVGYWHNWCDGA..GYKGGNAPCVTLDEIDPMYNVVNVSFMKVFN
consensus >70 .$k.k....a.....v..g........an..........v.vgywhnW..g...gy..gn.p...l.evn..Ynvvn!sfmk...
η2 η3 α1 β5 TT α2 β6 α3
80 90 100 110 120 130 140 150 160
CAM88673.1 IAEG..RIPTFKLDPTIALSEAEFIAQIDTLNSQGRSVLIALGGADAHI...ELTRGDEDALAAEIIRLTDLYGFDGLDIDLEQAAITAK
AAC46383.1 VADG..RIPTFKLDPTVGLSEEQFIDQVSELNKQGRSVLLALGGADAHV...ELETGDERAFADEIIRLTERYGFDGLDIDLEQAAVTAA
AAC09387.1 .GQG...IPTFK..PYNLSDAEFRRQVGVLNSQGRAVLISLGGADAHI...ELKTGDEDKLKDEIIRLVEVYGFDGLDIDLEQAAIGAA
AAO80224.1 .SDGTTRIPTFK..PYNQTDTAFRQEVAQLNSQGRAVLLALGGADAHI...QLVKGDEQAFANEIIRQVETYGFDGLDIDLEQLAITAG
BBA34114.1 .KDESTRNEDIR.....RALEQVLKRLVRSDAMQSKAVGAMLEKPEQFVLQYEYISKPSADTPLDYLRVD..FDSPRLNDALRKSNISPW
Chi1557 TSEG..RIPTFKLDPNIGLSEQQFIDQIGALNQQGRAVLIALGGADAHV...ELKTGDEQAFAQEIIRLTDKFGFDGLDIDLEQSAVTAS
Chi4668 TSEG..RIPTFKLDPNIGLSEQQFIDQISALNQQGRAVLIALGGADAHV...ELKTGDEQAFAQEIIRLTDKFGFDGLDIDLEQSAVTAS
consensus >70 ..#g..riptfk......ls#.qf..q!..ln.QgraVl.aLgga#ah!...#l..gdeqa.ad#iiRl.d.%gfdgL#idLeq.a!ta.
α4 β7 η4 TT α5 β8 TT β9 ******* β10 TT η5
170 180 190 200 210 220 230 240
CAM88673.1 D NQFVIPAALKMVKEHYRKTGDNFMITMAPEFPYLTANGAYTPYLTELDGYYDFINPQFYNQGGDGLWIEGV.GWIAQNNDALKEEFI
AAC46383.1 N NQTVIPDALKLVKDHYRAEGKNFLITMAPEFPYLTTGGKYVPYIDNLEGYYDWINPQFYNQGGDGIWVDGV.GWIAQNNDELKEEFI
AAC09387.1 N NKTVLPAALKKVKDHYAAQGKNFIISMAPEFPYLRTNGTYLDYINALEGYYDFIAPQYYNQGGDGIWVDELNAWFTQNNDAMKEDFL
AAO80224.1 D NQTVIPATLKIVKDHYRAQGKNFIITMAPEFPYLKPGAAYETYITSLNGYYDYIAPQLYNQGGDGVWVDEIMTWVAQSNDALKYEFL
BBA34114.1 G EQR..PEVLVWLSIEENSEKSIFAADQMPEMDRTLRAAAGENGLPVTAPLWDLTDQQNLNFE.........DFETGNGERIRQASA
Chi1557 D NQTVIPAALRLVKEHYQQQGKNFLITMAPEFPYLTEGGKYVPYITGLEGYYDWINPQFYNQGGDGIWVEGV.GWIAQNNDALKQEFI
Chi4668 D NQTVIPAALRLVKEHYQQQGKNFLITMAPEFPYLTEGGKYVPYITGLEGYYDWINPQFYNQGGDGIWVEGV.GWIAQNNDALKQEFI
consensus >70 d #qtviPaaL..vkehy..qgknF.itmaPEfpyl...g.y..yi..legyyD.inpQfyNqggdgiwve.v..wiaqnn#alkeef.
α6 η6 β11 α7 β12 α8 TT α9
250 260 270 280 290 300 310 320 330
CAM88673.1 IYYIADSLINGTRNYHKIPHDKLVFGLPSNIDAA ATGYIQDPQDLYKAFDRLKAQGQPLRGVMTWSVNWDMGTDAANNSYNQQFIKDYG
AAC46383.1 IYYISDSLINGTRGFHKIPHDKLVFGIPSSIDAA ATGFVKEPQDLYDAFDSLTAQGQPLRGVMTWSINWDMGTNKAGQAYNEQFIKDYG
AAC09387.1 LYYLTESLVTGTRGYAKIPAAKFVIGLPSNNDAA ATGYVIDKQAVYNAFARLDAKNLSIKGLMTWSINWDNGKSKAGVAYNWEFKTRYA
AAO80224.1 LYYMSDSLIHGTRGYLQIPNDKLVLGLPANRDAA GSGYVVEATPVAKTFDQLAKDGNPIRGLMTWSANWDVGQDVNGKSYNNEFATRYS
BBA34114.1 ARYESDAVLAG.....RLSH.........DAA ......EPDGTWDAVWRLYLQNIEET........WQGNSPDAPAAIRSGIAGVYS
Chi1557 IYYISDSLSNGTRGFHKIPHDKLVFGIPSNIDAA ATGFVQDPQDLYDAFDQLKAQGQALRGVMTWSVNWDMGTDKNGQEYGEKFVKDYG
Chi4668 IYYISDSLSNGTRGFHKIPHDKLVFGIPSNIDAV ATGFVQDPQDLYDAFDQLKAQGQALRGVMTWSVNWDMGTNKNGQAYGEKFVKDYG
consensus >70 .yY.s#sl..Gtrgy.kiphdklv.g.psn.daa atgyv.#pq..ydafd.L.aqgq..rg.mtwsvnW#.g.d...g..y.e.f...Y.
β13 β14 TT β15 TT η7 β16 TT β17 TT β18
340 350 360 370 380 390 400 410 420
CAM88673.1 NFIHNQL.PPVTDMTPTLSGIVDTRVELDSHFDPLIGITAKDYQGNDITADVTVSGSVNTNQVGYLLTYSV.SSDDETTNQPRKITVYE
AAC46383.1 PFVHGQVTPPPVEGEPMLKGVENTRVLHGTVFDPMEGETATDKEDGDLTSSIDVEGYVETSVIGYVLTYRVKDSDNNETTKARTVEVYS
AAC09387.1 PLIQGGVTPPP.............................GKPNAPTDVDG.............................
AAO80224.1 NLVK.............................
BBA34114.1 RLAE.............................
Chi1557 PFIHGQTPPPPSEGEPVLSGISDVRVHHGSSFDPYAGVTASDKEDGDLTNSITVEGSVDVNTVGYVLVYSVKDSDNNETKQSRTVVVYS
Chi4668 PFIHGQTPPPPSEGEPVFSGINDVRVHHGSAFDPYAGVTASDKEDGDLTNSITVEGSVDVNTVGYVLVYSVKDSDNNETKQSRTVVVYS
consensus >70 ..l.....PP.............................v.g.............................
β13 β14 TT β15 TT η7 β16 TT β17 TT β18
340 350 360 370 380 390 400 410 420
CAM88673.1 NFIHNQL.PPVTDMTPTLSGIVDTRVELDSHFDPLIGITAKDYQGNDITADVTVSGSVNTNQVGYLLTYSV.SSDDETTNQPRKITVYE
AAC46383.1 PFVHGQVTPPPVEGEPMLKGVENTRVLHGTVFDPMEGETATDKEDGDLTSSIDVEGYVETSVIGYVLTYRVKDSDNNETTKARTVEVYS
AAC09387.1 PLIQGGVTPPP.............................GKPNAPTDVDG.............................
AAO80224.1 NLVK.............................
BBA34114.1 RLAE.............................
Chi1557 PFIHGQTPPPPSEGEPVLSGISDVRVHHGSSFDPYAGVTASDKEDGDLTNSITVEGSVDVNTVGYVLVYSVKDSDNNETKQSRTVVVYS
Chi4668 PFIHGQTPPPPSEGEPVFSGINDVRVHHGSAFDPYAGVTASDKEDGDLTNSITVEGSVDVNTVGYVLVYSVKDSDNNETKQSRTVVVYS
consensus >70 ..i.....pp.............................v.g.............................
β19 β20 TT β21 TTT β22 η8 β23 TT β24 η9 β25
430 440 450 460 470 480 490 500
CAM88673.1 ILPAFTGITDTTVVIDSEFDPMQGVSASRPTQGDLTANITVTGEVDANVVGVYELTYQLFYGQDNQQNMTDKRIVTVVTDA.......
AAC46383.1 QKPVFDGVSDTTVVLGNSFDPMAGVTANDAEDGDLTSSIIHTGSVDVNEIGNYTLVYRV..TDSANQTVTAERKVTVTDGSNCAAAWD
AAC09387.1 ...RRAGRHLAETKLGRRHRALPIASYTVYRNGNPIGGQTAGLSLTDSGLTPATQYSYFVAATDSQGNTSLPSSALAVKTATD......
AAO80224.1 ....
BBA34114.1 ...RFIPRTTRETILELKVFGLSSLGAIDRAASNLSGLSQVRKLEMLGVDADYALFKLYVRGDRASLEETLALGRVLRPVA.......
Chi1557 LVPEFEGVADTTIQLGDAFDPPMAGVKATDAEDGDLTDQVRVEGSVDVNVLGVYDLVYRV..TDSANQTTAQRSVIVSDGS.......
Chi4668 LVPELEGVADTTIQLGDAFDLLMAGVKATDAEDGDLTDQVRVEGRVDVNVLGVYNLVYRV..TDSANQTTAQRSVIVSDGS.......
consensus >70 .....g.......l......m....a....dgdl.........d......y.l.y.....d...q..t......v........
CAM88673.1 ....
AAC46383.1 ANTVYVEGDQVSHDGATWVAGWYTRGEEPGTTGEWGVWKKASDSSCGGNPSPGGDVELSVSGLQSILDNGDVRIQFTLASNEALDVIAK
AAC09387.1 ....
AAO80224.1 ....
BBA34114.1 ....
Chi1557 ....
Chi4668 ....
consensus >70 ....
TT β26 TT β27 TT β28
510 520 530
CAM88673.1 .............................VSDDDWQVGSTYVKDDKVT.HNGATWT
AAC46383.1 VINSAGSVVEQTNVNLTDSRTVTMDLYDVTEGQYKEVVGTATDGEVVMVDNSFSVKEEGGTTPPPGNYPPYAAGTNYEAGDIVVGNDNGLYE
AAC09387.1 .............................GTPPDPGAPEWQNNHSYKAGDVVS.YKGKKYT
AAO80224.1 ....
BBA34114.1 .............................NEDRQFSGLTYQLAE.......
Chi1557 .............................SYPPYESGKTYEAGDIVTGSDGNLYQ
Chi4668 .............................SYPPYESGKAYEAGDIVTGSDGNLYQ
consensus >70 ....g..y...d.v........

Figure S5. Multiple sequence alignment of the amino acid sequences of Chi1557, Chi4668 and other family GH18 chitinases from different bacterial by MUSCLE program in the MEGA version 7.0 and enhanced by ESPript v3.0. *, the characteristic motifs *DxDxDxE* were the signature of chitinases from family GH18. α, α-helix; β, β-helix fold; η, random coil.

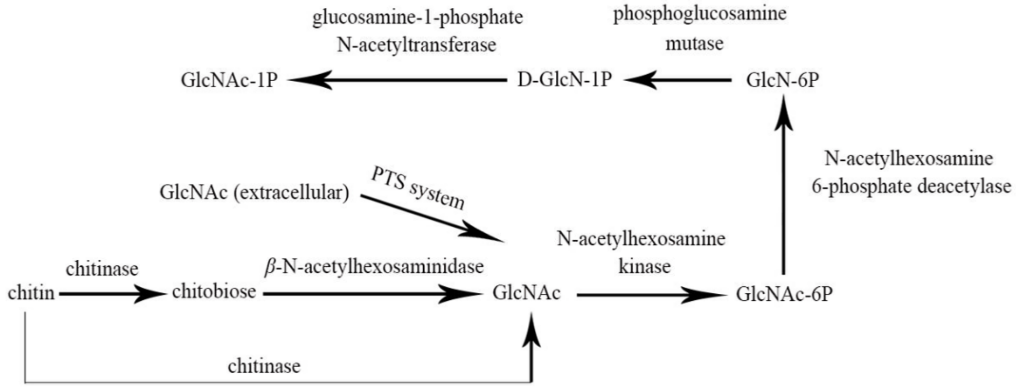

Figure S6. The chitin metabolic pathway in *Vibrio rotiferianus* WXL191 and *V. harveyi* WXL538 annotated by KAAS (KEGG Automatic Annotation Server).
